# Advances in mechanisms of neuroplasticity induced by multimodal closed-loop brain–computer interfaces after stroke

**DOI:** 10.3389/fnhum.2026.1828191

**Published:** 2026-06-02

**Authors:** Yufeng Chen, Yongmei Jiang, Xiran Wang, Miao Zeng, Jiahao Cui

**Affiliations:** 1 College of Health and Rehabilitation Sciences, Dalian Medical University, Dalian, Liaoning, China; 2 The Second Affiliated Hospital of Dalian Medical University (Diamond Bay Campus), Dalian, Liaoning, China

**Keywords:** brain–computer interface, functional electrical stimulation, Hebbian learning, motor intent decoding, multimodal feedback, neuroplasticity, stroke rehabilitation, virtual reality

## Abstract

Post-stroke motor dysfunction is one of the leading causes of acquired disability worldwide. The induction and maintenance of neuroplasticity constitute the core mechanisms underlying motor function recovery. Conventional open-loop brain–computer interfaces (BCIs) lack real-time closed-loop feedback and are therefore unable to reliably activate the “temporal contingency” principle required by Hebbian synaptic remodeling, resulting in limited rehabilitation efficacy. Multimodal closed-loop BCIs integrate motor intent decoding, functional electrical stimulation (FES), virtual reality (VR), and exoskeleton-mediated proprioceptive feedback to construct a complete sensorimotor closed-loop circuit. These systems can precisely induce activity-dependent synaptic plasticity, facilitate cortical reorganization, and ameliorate interhemispheric inhibitory imbalance. The present review systematically examines the theoretical foundations of neuroplasticity induction by multimodal closed-loop BCIs following stroke, the constituent system components, electrophysiological and neuroimaging evidence, and the key factors modulating neuroplasticity induction efficacy. Future directions toward personalized adaptive closed-loop systems and long-term home-based rehabilitation are discussed. This review integrates converging evidence from electroencephalography, functional magnetic resonance imaging, transcranial magnetic stimulation, and randomized controlled trials to establish a comprehensive mechanistic framework for multimodal BCI-mediated neuroplasticity, and provides reference for both basic research and clinical translation in this field.

## 1 Introduction

### 1.1 Neural mechanisms of post-stroke motor impairment

Stroke is the second leading cause of death globally and the primary cause of acquired motor disability in adults. Epidemiological data indicate that approximately 15 million new stroke cases occur annually worldwide, of which roughly 75% of survivors experience residual motor deficits of varying severity, substantially impairing activities of daily living and quality of life (Mane et al., 2020). Ischemic or hemorrhagic stroke damages cortical and subcortical motor networks, producing contralateral hemiplegia or incomplete paresis. Although neuronal death within the lesion core is irreversible, the peri-infarct penumbra and remote regions retain plasticity potential and thus constitute the neurobiological substrate for motor function recovery (Azad et al., 2016).

In the acute phase following stroke, excitatory imbalance between the ipsilesional primary motor cortex (M1) and the contralesional hemisphere is markedly exacerbated. The ipsilesional M1 exhibits reduced excitability, while the contralesional M1 is overactivated and exerts transcallosal inhibition (TCI) on the ipsilesional side, creating a pathological feedback loop (Gao et al., 2022). This “interhemispheric imbalance” mechanism is widely regarded as one of the core pathological drivers of diminished corticospinal tract (CST) output and persistent motor impairment (Krucoff et al., 2016).

### 1.2 Neuroplasticity as the foundation of rehabilitation

Neuroplasticity refers to the capacity of the nervous system to repair and adapt through changes in synaptic strength, axonal sprouting, and functional cortical remapping in response to internal and external environmental demands (Oweiss and Badreldin, 2015). Extensive animal and human studies confirm that meaningful sensorimotor experience can induce activity-dependent synaptic plasticity, enabling surviving motor neurons connected to the affected limb to re-establish effective corticospinal synaptic connections (Sitaram et al., 2017).

The Hebbian learning principle—“neurons that fire together wire together”—provides the classical theoretical framework for activity-dependent plasticity: when motor intent signals (cortical discharge) and sensory afferent feedback (proprioception, tactile, and visual inputs) are precisely synchronized in time, synaptic efficacy undergoes sustained enhancement, i.e., long-term potentiation (LTP) (Krueger et al., 2024). This principle directly determines the core design imperative of BCI rehabilitation systems: feedback must be temporally synchronized with motor intent to effectively induce neuroplasticity.

### 1.3 Limitations of open-loop BCIs and the emergence of multimodal closed-loop systems

Brain–computer interfaces decode neural signals—primarily electroencephalography (EEG)—in real time and translate them into control commands for external devices, providing a novel avenue for patients with motor disorders to participate actively in rehabilitation training (Ushiba and Soekadar, 2016; Tonin et al., 2025). Early BCI systems adopted open-loop architectures in which EEG signals triggered predefined visual feedback or mechanical stimulation without dynamic adjustment based on the patient’s current neural state, thus providing insufficient guarantee of sensorimotor temporal contingency and limiting the efficiency of neuroplasticity induction (Le Franc et al., 2022).

The concept of multimodal closed-loop BCIs has consequently emerged: the system continuously monitors the patient’s EEG, recognizes motor intent in real time, and synchronously triggers multiple sensory feedback modalities upon intent detection—including FES-evoked muscle contraction (proprioceptive and tactile), limb visual–motor feedback within VR environments, and exoskeleton-assisted mechanical movement—thereby precisely activating the multisensory integration required for Hebbian synaptic remodeling in time and space (Saway et al., 2024; Liu et al., 2025).

**Figure 1:**
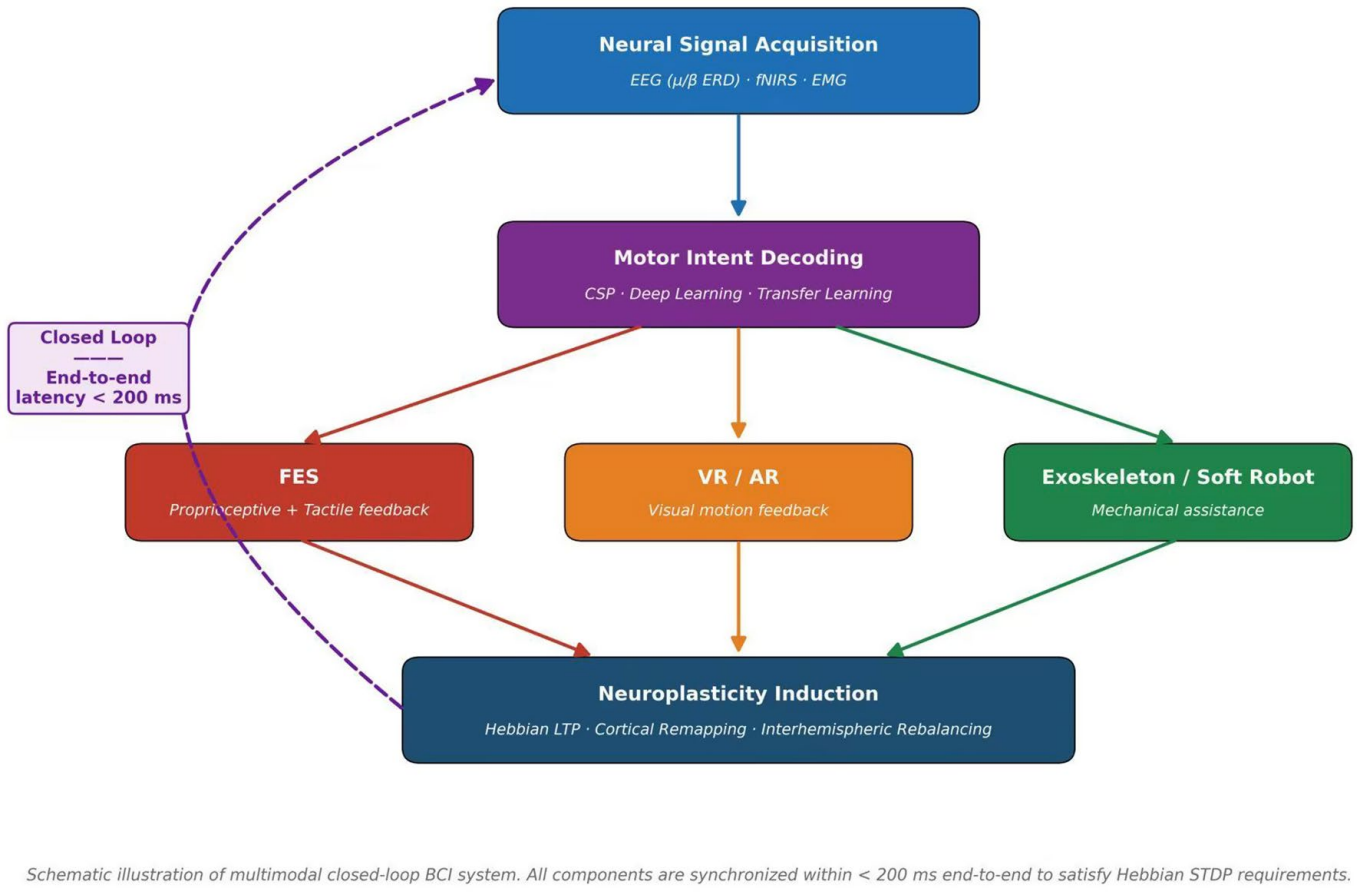
Multimodal closed-loop BCI system architecture for post-stroke neuroplasticity induction.

The present review systematically examines the theoretical foundations of multimodal closed-loop BCI-induced neuroplasticity following stroke, including engineering implementation and neurobiological evidence, and discusses key factors influencing therapeutic efficacy and future directions, with the aim of providing reference for basic research and clinical translation in this field. The complete architecture of a multimodal closed-loop BCI system for post-stroke neuroplasticity induction is illustrated in Figure 1.

### 1.4 Literature search strategy

The literature included in this review was identified through systematic searches of four major databases: PubMed, IEEE Xplore, Web of Science, and Scopus. Search terms included combinations of the following keywords: “brain-computer interface,” “BCI,” “stroke rehabilitation,” “neuroplasticity,” “motor imagery,” “functional electrical stimulation,” “virtual reality,” “exoskeleton,” “closed-loop,” “Hebbian learning,” “cortical reorganization,” and “sensorimotor rhythm.” The search covered publications from January 2015 to March 2026, with the exception of seminal foundational works cited for theoretical context, which were included regardless of publication date. Inclusion criteria were: (1) peer-reviewed journal articles or conference proceedings; (2) studies involving human participants or directly relevant to human BCI rehabilitation; (3) studies reporting neuroplasticity indices, motor function outcomes, or BCI decoding performance. Exclusion criteria were: (1) non-English publications for which no translation was available; (2) conference abstracts without accompanying full-text data; (3) studies exclusively involving non-stroke neurological populations without direct relevance to stroke rehabilitation. This review is intended as a narrative review with a structured search strategy, consistent with the Review Article format of Frontiers in Human Neuroscience, and does not follow the PRISMA protocol for systematic reviews.

## 2 Core mechanisms of neuroplasticity

### 2.1 Hebbian learning and activity-dependent synaptic remodeling

Hebbian synaptic plasticity theory, originally proposed by Donald Hebb in 1949, finds its modern biological correlate in spike-timing dependent plasticity (STDP), which specifies that LTP occurs when presynaptic neuronal firing precedes postsynaptic firing by a few milliseconds; the reverse temporal order produces long-term depression (LTD) (Sitaram et al., 2017). This principle has direct implications for BCI system design: if the decoding latency of motor intent detection exceeds the plasticity time window of approximately 100–200 ms following cortical discharge, the feedback will fail to effectively activate Hebbian mechanisms (Mrachacz-Kersting et al., 2016; Niazi et al., 2022).

Mrachacz-Kersting et al. (2016) designed a landmark “associative BCI” experiment in which the BCI precisely detected the negative peak of the movement-related cortical potential (MRCP) during ankle dorsiflexion motor intent and triggered peroneal nerve electrical stimulation approximately 100 ms post-peak, thereby satisfying STDP temporal requirements. After only 50 temporally paired events, chronic stroke patients demonstrated significant improvements in dorsiflexor muscle strength and increases in cortical motor evoked potential (MEP) amplitude, confirming the central role of temporal precision in neuroplasticity induction (Mrachacz-Kersting et al., 2016).

**Figure 2:**
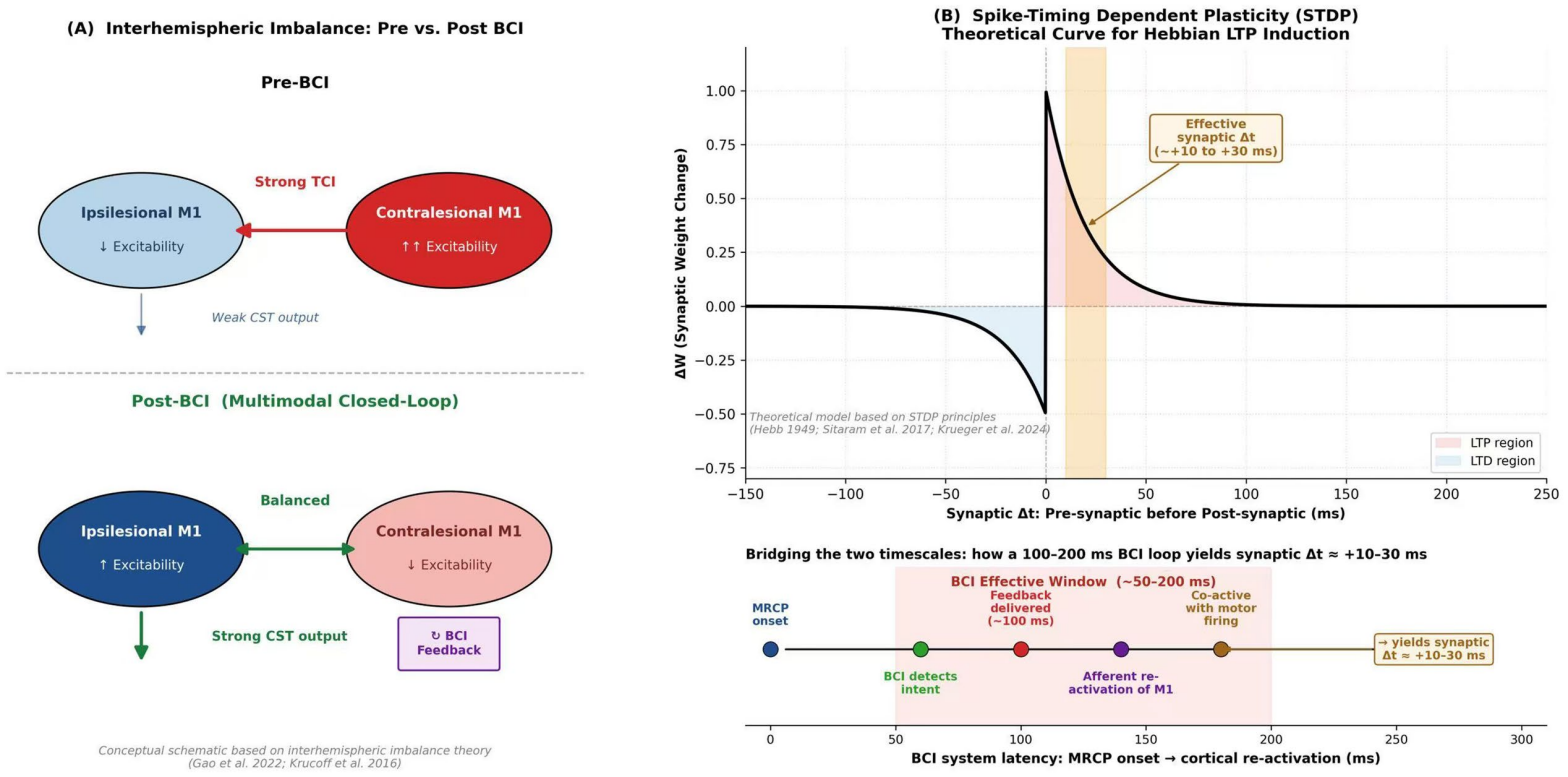
Neuroplasticity mechanisms underlying multimodal closed-loop BCI rehabilitation.

Krueger et al. (2024) subsequently provided direct validation of the relationship between Hebbian plasticity and BCI intervention in human stroke patients, demonstrating that temporally contingent sensory feedback not only augmented motor evoked potentials but also correlated significantly with the degree of cortical reorganization. These findings collectively establish “temporal synchrony of feedback” as a core engineering design constraint for multimodal closed-loop BCIs. The STDP theoretical curve and its relationship to the BCI effective timing window are illustrated in Figure 2B. The interhemispheric imbalance mechanism and its correction through multimodal closed-loop BCI intervention are schematically depicted in Figure 2A.

### 2.2 Cortical reorganization and interhemispheric inhibitory imbalance

Following stroke, reduced excitability of the lesioned hemisphere’s M1 and sustained transcallosal inhibition from the contralesional M1 generate a “competitive imbalance.” Neuroimaging studies consistently demonstrate that patients with favorable functional recovery exhibit a gradual restoration of ipsilesional M1 excitability and a trend toward interhemispheric competitive equilibrium (Yuan et al., 2020; Caria et al., 2020).

Modulation of interhemispheric imbalance can be achieved through two complementary strategies: (i) enhancement of activity-dependent plasticity in the ipsilesional M1 through BCI-guided active motor intent training to elevate cortical excitability; and (ii) suppression of excessive contralesional M1 activation, achievable through 1 Hz repetitive transcranial magnetic stimulation (rTMS) of the contralesional hemisphere or bilateral training paradigms (Chew et al., 2020; Hong et al., 2017). Multimodal closed-loop BCIs, by simultaneously activating multiple afferent modalities within the ipsilesional sensorimotor cortex, are considered a highly efficient means of implementing strategy (i). Jia et al. (2025) further demonstrated that individual differences in cortical activation patterns—specifically whether ipsilesional cortical activation or ipsilesional inhibition is predominant—determine the optimal rTMS target selection for combined rTMS–BCI therapy, underscoring the importance of personalizing interhemispheric modulation strategies.

Functional connectivity analyses confirm that post-BCI intervention the internal functional connectivity of the ipsilesional motor network is significantly enhanced, manifesting as increased synchrony between M1 and the supplementary motor area (SMA) and parietal cortex (Grigoryan et al., 2025; Lau et al., 2021). These electrophysiological and neuroimaging indices of cortical reorganization form the core experimental foundation for understanding the neuroplasticity mechanisms of multimodal closed-loop BCIs.

### 2.3 Sensorimotor integration and the role of proprioceptive feedback

Sensorimotor integration refers to the process by which the cerebral cortex matches proprioceptive afferent signals from muscle spindles, cutaneous mechanoreceptors, and joint receptors with internal motor commands and corrects for prediction errors. This process is primarily mediated by the sensory cortex (S1) and parietal areas 5 and 7 (Jeunet et al., 2019). Effective sensorimotor integration is critical for maintaining corticospinal synaptic efficacy; post-stroke S1 damage or reduced sensory afference further diminishes the plasticity potential of the motor network.

Mrachacz-Kersting et al. (2017) systematically compared the cortical plasticity effects of different afferent feedback modalities—electrically evoked proprioception versus median nerve stimulation producing mixed sensation—and found that muscle proprioceptive afference (corresponding to Ia fiber activation of muscle spindles), when synchronized with the motor intent signal, produced the strongest cortical plasticity effects, whereas pure cutaneous stimulation yielded comparatively weaker outcomes. Jochumsen et al. (2018, 2019) further confirmed that the selection of the optimal afferent feedback modality exerts a decisive influence on the efficiency of neuroplasticity induction in self-paced BCI paradigms, with substantial individual variability across modalities.

VR-mediated visual–motor feedback can activate the frontoparietal mirror neuron system (MNS), augmenting motor observation-induced cortical desynchronization (event-related desynchronization, ERD) (Vourvopoulos et al., 2019), while vibrotactile stimulation directly activates cutaneous mechanoreceptors and muscle spindles, enhancing periodic firing patterns of the sensory cortex and improving the signal-to-noise ratio of EEG (Shu et al., 2019). The integration of these multimodal afferent inputs constitutes the neurobiological foundation for closed-loop BCI-induced sensorimotor plasticity.

## 3 System components of multimodal closed-loop BCIs

### 3.1 Signal acquisition: EEG-centered multi-modal recording

EEG is currently the most widely employed neural signal acquisition modality in stroke rehabilitation BCIs, offering non-invasiveness, high temporal resolution (millisecond scale), and relatively low cost.

The primary functional signals acquired include the movement-related cortical potential (MRCP) and sensorimotor rhythms (SMR).

The MRCP is a slow negative cortical potential that emerges approximately 1–2 s prior to voluntary movement onset, reflecting the sequential activation of the supplementary motor area (SMA), premotor cortex (PMC), and primary motor cortex (M1). Its most diagnostically relevant component is the Bereitschaftspotential (BP), whose negative slope—maximal at electrode positions Cz and FCz—serves as the primary BCI trigger window. The temporal precision with which the MRCP negative peak is detected is critical for STDP-based neuroplasticity induction: as established in Section 2.1, feedback delivered approximately 100 ms post-peak satisfies the Hebbian temporal contingency requirement, whereas detection latencies exceeding 200 ms fall outside the effective plasticity window (Mrachacz-Kersting et al., 2016). In stroke patients, MRCP amplitude is typically attenuated and spatially diffuse owing to disrupted corticospinal tract integrity, making reliable detection a significant signal processing challenge.

Sensorimotor rhythms (SMR) refer to oscillatory EEG activity in the mu (8–13 Hz) and beta (13–30 Hz) frequency bands generated by the sensorimotor cortex. Event-related desynchronization (ERD)—a power decrease in these bands—occurs during movement preparation and execution, as well as during motor imagery, reflecting increased cortical excitability and neural engagement. Conversely, event-related synchronization (ERS)—a power increase—follows movement completion and is associated with cortical inhibition and sensorimotor gating. In BCI rehabilitation, ipsilesional ERD amplitude and its spatial focalization toward the contralateral motor cortex are established electrophysiological markers of neuroplasticity reorganization: increased ipsilesional ERD following BCI intervention reflects enhanced recruitment of the lesioned hemisphere’s motor network (Remsik et al., 2019; Gangadharan et al., 2024).

Regarding experimental paradigms, a distinction must be drawn between cue-based and self-paced BCI protocols. The Graz paradigm—developed at the Technical University of Graz and widely adopted as a benchmark protocol—employs a cue-based, trial-structured design in which visual or auditory cues instruct the participant to perform motor imagery during clearly defined active periods, separated by standardized rest intervals. This structure facilitates reliable ERD/ERS feature extraction and classifier training, and forms the basis for many benchmark datasets including BCI Competition IV. However, the cue-driven nature of the Graz paradigm limits its ecological validity for clinical rehabilitation, as real-world motor recovery requires volitional, self-initiated movement attempts rather than externally prompted imagery. Self-paced BCI paradigms, which detect spontaneous motor intent without external cueing, better preserve the authentic temporal structure of motor intention and have been shown to induce more stable LTP-like cortical plasticity effects (Jochumsen et al., 2013), though they impose substantially greater demands on decoding algorithm sensitivity and specificity (Tables 1–3).

**Table 1:** Comparison of representative motor intent decoding algorithms for BCI rehabilitation.

Algorithm	Accuracy	Condition	Latency	Cross-subject	Clinical applicability
CSP + LDA	~80%	Offline, healthy	Low	Poor	Limited
Riemannian geometry	81.75%	Online, stroke patients	Low-Medium	Moderate	Good
EEGNet	~85%	Offline, healthy	Very low	Moderate	Promising
Swin transformer	87.67%	Offline, healthy	High	Moderate	Needs validation
IFNet	~82% online	Online, healthy	Medium	Good	Promising
MI-MBFT	86.93%	Offline, healthy	High	Moderate	Needs validation
ONCS + transfer	Comparable to repeated calibration	Online, healthy	Low	Good	Promising

Offline results on benchmark datasets should not be directly compared with online clinical results. CSP, common spatial pattern; LDA, linear discriminant analysis; ONCS, once-calibration strategy.

**Table 2:** Systematic comparison of multimodal feedback modalities in closed-loop BCI rehabilitation.

Dimension	FES	VR	Exoskeleton/soft robot
Primary afferent pathway	Proprioceptive (Ia fibers)	Visual-motor (mirror neuron system)	Proprioceptive + tactile + kinesthetic
Neuroplasticity evidence level	Strong (multiple RCTs)	Moderate (feasibility studies)	Moderate (RCTs with active BCI trigger)
Key mechanism	Peripheral STDP via Ia afferent activation	Mirror neuron-mediated ERD augmentation	Multisensory integration via broad afferent activation
Clinical practicality	High	Moderate	Low-Moderate
Patient burden	Low	Moderate	High
Home deployment feasibility	High	Moderate	Low (improving)
Representative evidence	Biasiucci et al. (2018), Chen et al. (2025)	Vourvopoulos et al. (2019), Wang et al. (2025b)	Cheng et al. (2020), Premchand et al. (2025)

FES, functional electrical stimulation; VR, virtual reality; RCT, randomized controlled trial; STDP, spike-timing dependent plasticity; ERD, event-related desynchronization.

**Table 3:** Summary of key quantitative neuroplasticity evidence from representative BCI intervention studies.

Study	Design	Population	Neuroplasticity index	Key finding	Clinical outcome
Biasiucci et al. (2018)	RCT	Chronic stroke (*n* = 19)	fMRI ipsilesional M1 activation	Significant post-treatment increase in ipsilesional M1 activation	FMA-UE improved; sustained at 12-week follow-up
Mrachacz-Kersting et al. (2016)	Controlled trial	Chronic stroke	TMS MEP amplitude	Significant MEP increase after 50 paired events	Significant dorsiflexor strength improvement
Jochumsen et al. (2019)	Controlled trial	Healthy subjects	TMS MEP amplitude	EEG-triggered: +62% immediately, +72% at 30 min; EMG-triggered: +73%, +79%	N/A (healthy subjects)
Vourvopoulos et al. (2019)	Feasibility study	Chronic stroke	fMRI motor network activation	Significant ipsilesional motor network enhancement post-VR-BCI	Functional improvement trend
Cheng et al. (2020)	RCT	Chronic stroke	FMA, ARAT	Persistent improvement at 12- and 24-week follow-up in BCI-SRG group; none in robot-only group	FMA and ARAT sustained at follow-up
Shu et al. (2019)	Controlled trial	Healthy subjects	EEG MRCA; online BCI accuracy	TS-MA: MRCA higher than MA or TS alone; accuracy 74.5% (MA) vs. 85.1% (TS-MA)	N/A (healthy subjects)
Krueger et al. (2024)	Controlled trial	Stroke patients	TMS MEP; cortical reorganization	Contingent feedback augmented MEP; significant correlation with cortical reorganization	Motor recovery improvement
Rodríguez-García et al. (2025)	Prospective study	Chronic stroke	Cortical activity; GM volume; WM FA	GM thickening around ipsilesional motor area; CST FA increase	Multi-level structural-functional plasticity
Yuan K. et al. (2021)	RCT	Subacute stroke	fMRI M1-SMA connectivity	Significant enhancement of ipsilesional M1-SMA connectivity	FMA improvement correlated with connectivity
Cervera et al. (2018)	Meta-analysis	Stroke (*n* = 476)	FMA-UE	Overall SMD = 0.53; FES and high dose as independent predictors	Significant upper-limb functional improvement
Zhang et al. (2025)	Network meta-analysis	Stroke	FMA-UE	MI-BCI + FES + tDCS ranked highest	Triple combination superior to unimodal/dual
Chen et al. (2025)	Longitudinal follow-up	Elderly stroke	fMRI activation; connectivity	Continuous cortical expansion and stable connectivity over 4.5 years	Sustained neuroplasticity and motor function

RCT, randomized controlled trial; FMA-UE, Fugl-Meyer Assessment Upper Extremity; ARAT, Action Research Arm Test; MEP, motor evoked potential; FA, fractional anisotropy; CST, corticospinal tract; SMD, standardized mean difference; GM, gray matter; WM, white matter; MRCA, motor-related cortical activation; TS-MA, tactile stimulation plus motor attempt.

Multi-channel EEG systems (64–256 channels) afford rich spatial information but impose high impedance preparation overhead, creating practical barriers for clinical deployment. Recent research has therefore pursued low-channel wearable EEG solutions: Rao et al. (2024) developed a 4-channel wireless headband-based wearable motor imagery (MI) BCI that achieved 85.21% offline classification accuracy and 76.54% online accuracy across 66 healthy participants, comparable to established commercial systems. Chowdhury et al. (2026) conducted online validation in real-world noisy environments using an 8-channel g.tec Unicorn headband, providing the Neurestore benchmark dataset as a critical reference for wearable BCI clinical deployment.

To compensate for EEG’s limited spatial resolution, multimodal neural recording approaches have gained traction. Functional near-infrared spectroscopy (fNIRS) provides hemodynamic information that is temporally and spatially complementary to EEG: Ji et al. (2025) simultaneously acquired EEG and fNIRS in a randomized controlled trial (RCT) with subacute stroke patients, enabling dual-modality monitoring of motor intent and cortical oxygenation. Zhang et al. (2019) fused EEG, EMG, and EOG tri-modal signals to construct a multimodal human–machine interface for soft robotic hand control, substantially improving system robustness and usability. Concurrent EMG and EEG acquisition has also been explored for corticomuscular coherence (CMC) analysis: Chowdhury et al. (2020) demonstrated that cortical–muscular co-activation features (CBPT) can serve both as BCI control signals and as biomarkers of motor recovery monitoring.

### 3.2 Decoding layer: intelligent motor intent recognition

Motor intent decoding is the core computational stage of multimodal closed-loop BCIs; its accuracy and latency directly determine the temporal synchrony of feedback and, consequently, the efficiency of neuroplasticity induction. Before reviewing specific algorithms, it is important to distinguish three categories of experimental evidence that carry fundamentally different levels of clinical relevance: (1) offline classification on benchmark datasets (e.g., BCI Competition IV Dataset 2a), which provides a standardized basis for algorithmic comparison but is susceptible to overfitting and does not reflect real-time constraints; (2) online classification in healthy subjects, which introduces real-time processing demands but does not capture the signal degradation characteristic of stroke patients; and (3) online classification in stroke patients, which is the only condition with direct clinical validity. High offline accuracy does not reliably predict online clinical performance owing to the domain shift problem—the systematic difference between the statistical distributions of training data and real-world patient EEG signals—and results should be interpreted accordingly.

Traditional approaches extract frequency-band power features (mu/beta ERD) combined with the common spatial pattern (CSP) algorithm for binary classification, achieving approximately 80% accuracy in healthy subjects under offline conditions. However, in stroke patients, classification performance is substantially degraded owing to attenuated and spatially diffuse neural signals, high inter-individual variability, and non-stationarity across sessions (Gaur et al., 2021; Ang and Guan, 2017).

Deep learning methods have markedly improved MI decoding performance. Wang et al. (2025a) proposed IFNet (interactive frequency convolutional neural network), achieving approximately 20–27% improvement over FBCSP baselines in online 2-class continuous control tasks in healthy subjects, with cross-session transfer demonstrated—the first such demonstration for deep learning in online MI-BCIs. Wang et al. (2023) proposed a channel attention combined Swin Transformer achieving 87.67% accuracy in multi-class MI classification under offline conditions on BCI Competition IV. Luo et al. (2024) achieved 86.93% offline accuracy on BCI Competition IV Dataset 2a using MI-MBFT. It should be noted that these results were obtained under offline or healthy-subject conditions; their generalizability to online stroke patient data requires further validation.

Transfer learning represents an important strategy for addressing the high calibration cost of BCIs. Rao et al. (2025) introduced a once-calibration strategy (ONCS) combined with supervised transfer learning allowing continuous BCI interventions spanning up to 1 month with only a single calibration session in healthy subjects, maintaining accuracy comparable to repeated calibration. Gaur et al. (2022) achieved 81.75% mean accuracy on a dataset of 10 hemiplegic stroke patients—an online, within-subject result with direct clinical relevance—using a tangent-space Riemannian geometry cross-subject transfer approach, demonstrating the practical feasibility of calibration-free BCIs in clinical populations. Zhang et al. (2023) proposed an adaptive BCI system that dynamically adjusts stimulation parameters based on real-time BCI performance in stroke patients, enabling patients with low BCI performance (LBP) to benefit from FES rehabilitation.

Beyond motor imagery classification, two additional decoding targets deserve explicit consideration for their clinical relevance. First, kinetic characteristics decoding—the estimation of movement parameters such as grip force, movement velocity, and joint torque from EEG signals—enables more nuanced and functionally graded motor control beyond binary intent detection, potentially supporting more naturalistic rehabilitation paradigms (Müller-Putz et al., 2022). Second, muscle activity decoding approaches leverage corticomuscular coherence (CMC) features derived from simultaneous EEG and EMG recordings to decode motor output at the level of individual muscle groups (Amiri and Shalchyan, 2025). These EMG-based approaches are particularly relevant for stroke patients with residual voluntary muscle activation, as they can exploit preserved corticospinal connections that may be invisible to EEG-only decoders. The integration of these alternative decoding targets with multimodal closed-loop feedback architectures represents an important direction for expanding the clinical applicability of BCI rehabilitation to patients across a wider spectrum of motor impairment severity (Yang et al., 2022).

### 3.3 Multimodal feedback execution

#### 3.3.1 Somatosensory feedback: functional electrical stimulation (FES)

Functional electrical stimulation activates motor nerves in the paralyzed limb via transcutaneous electrodes, producing passive joint movement and simultaneously activating muscle spindle Ia afferent fibers in temporal alignment with motor cortex discharge, thereby satisfying the temporal conditions of Hebbian STDP (Biasiucci et al., 2018). The RCT published by Biasiucci et al. (2018) represents a landmark in the BCI–FES field: 19 chronic stroke patients receiving BCI–FES treatment demonstrated significant improvements in the Fugl-Meyer Assessment—Upper Extremity (FMA-UE) score, with efficacy sustained at 12-week follow-up, indicating a maintained neuroplasticity effect. Concurrent fMRI assessment revealed significant post-treatment increases in ipsilesional M1 activation and enhanced functional connectivity.

Chen et al. (2025) published the longest-duration BCI–FES study to date (4.5-year longitudinal follow-up), demonstrating sustained neuroplasticity effects of long-term BCI–FES intervention in elderly stroke patients, including continuous expansion of cortical activation areas and stable enhancement of functional connectivity. Regarding the precision of EEG–FES temporal synchrony: Jochumsen et al. (2019) compared EEG-triggered versus EMG-triggered electrical stimulation in healthy subjects for inducing corticospinal plasticity. Both triggers produced significant MEP amplitude increases of approximately 62–73%, suggesting that stroke patients with residual EMG signals may use EMG signals as the trigger, simplifying system design.

#### 3.3.2 Visual feedback: immersive VR environments

Virtual reality technology provides BCIs with an immersive, quantifiable visual–motor feedback platform. Action observation (AO) within VR environments can activate the sensorimotor cortex through the mirror neuron system, inducing ERD patterns similar to actual movement and thereby augmenting BCI training outcomes without requiring actual movement execution (Vourvopoulos et al., 2019). Vourvopoulos et al. (2019) conducted a feasibility evaluation of a VR-BCI system in chronic stroke patients, with fMRI comparison of brain activation before and after treatment revealing significant enhancement of ipsilesional motor network activation in the VR-BCI group.

Wang et al. (2025b) proposed an adaptive neurofeedback training (NFT) system based on VR games combined with MI-BCI, improving classification accuracy by approximately 10% relative to the conventional Graz paradigm and reducing training duration by more than 30% through adaptive task difficulty modulation. Tang et al. (2023) integrated VR with exoskeleton and EEG decoding into a unified VR–ULE system, employing SE attention modules to individually optimize optimal frequency band feature weighting for different patients, achieving 86.49% classification accuracy and 86–88% task completion rate in upper-limb rehabilitation tasks.

#### 3.3.3 Proprioceptive feedback: exoskeletons and soft robotic devices

Exoskeletons and soft robotic gloves provide integrated proprioceptive and tactile feedback by physically assisting limb movement, representing the execution-end devices in multimodal closed-loop BCIs most capable of directly inducing sensorimotor integration. Cheng et al. (2020) conducted an RCT demonstrating that the BCI–soft robotic glove (BCI-SRG) group exhibited persistent trends of improvement in FMA and ARAT scores at follow-up assessments (12 and 24 weeks) after the 6-week intervention, whereas the robotic glove alone group showed no sustained effect, indicating that BCI-mediated active intent triggering is a necessary condition for long-lasting neuroplasticity.

Premchand et al. (2025) systematically described the complete architecture of a personalized multimodal BCI–soft robotic system incorporating MI decoding, FES somatosensory feedback, and coordinated soft finger exoskeleton control, and verified system feasibility in chronic stroke patients. Lu et al. (2025) published key mechanistic findings: multisensory BCI, while promoting motor recovery, ameliorated callosal fiber function through interhemispheric integration mechanisms, providing direct neuroimaging evidence for multimodal feedback-induced white matter plasticity. Zhang et al. (2026) found that vibrotactile stimulation significantly enhanced bilateral sensorimotor cortical activation in stroke patients—not limited to the contralateral cortex—suggesting that tactile stimulation may promote neuroplasticity through augmented interhemispheric integration.

#### 3.3.4 Comparative analysis of multimodal feedback modalities

The three principal feedback modalities employed in multimodal closed-loop BCIs—FES, VR, and exoskeleton/soft robotic devices—differ substantially in their neuroplasticity induction mechanisms, clinical practicality, and suitability for home-based deployment. In terms of neuroplasticity induction efficacy, FES currently holds the strongest evidence base. By directly activating motor nerves and muscle spindle Ia afferent fibers in temporal alignment with motor cortex discharge, FES satisfies the STDP temporal contingency requirement at the peripheral level, producing robust and sustained MEP amplitude increases and ipsilesional M1 activation (Biasiucci et al., 2018; Jochumsen et al., 2019). VR-mediated visual feedback activates the frontoparietal mirror neuron system, inducing ERD patterns similar to actual movement through action observation; however, its neuroplasticity effects are primarily mediated through visual rather than somatosensory pathways, and the evidence for sustained structural plasticity remains comparatively limited (Vourvopoulos et al., 2019). Exoskeleton and soft robotic devices provide the most comprehensive somatosensory input by physically assisting limb movement, simultaneously activating muscle spindles, cutaneous mechanoreceptors, and joint receptors; however, their neuroplasticity induction efficacy is contingent on BCI-triggered active intent, as passive mechanical assistance alone does not produce sustained plasticity effects (Cheng et al., 2020).

Regarding afferent pathway specificity, FES selectively targets the proprioceptive pathway via Ia afferent fiber activation, which has been shown to produce stronger cortical plasticity effects than cutaneous stimulation alone (Mrachacz-Kersting et al., 2017). VR engages the visual-motor pathway via the mirror neuron system, offering a complementary but distinct afferent channel. Exoskeletons and soft robots engage the broadest range of afferent pathways simultaneously, including proprioceptive, tactile, and kinesthetic channels, which may account for the superior neuroplasticity outcomes observed with multimodal combinations. In terms of clinical practicality, FES systems are the most clinically mature. VR systems require specialized displays but have improved substantially in accessibility. Exoskeletons remain the most expensive and technically demanding modality. Regarding home deployment feasibility, FES combined with low-density wearable EEG currently represents the most technically viable pathway for home-based multimodal BCI rehabilitation (Rao et al., 2025).

## 4 Evidence for neuroplasticity induction by multimodal closed-loop BCIs

### 4.1 Electrophysiological indices

Event-related desynchronization/synchronization (ERD/ERS) of the mu and beta rhythms are the most established EEG markers of sensorimotor cortical plasticity. ERD amplitude and spatial distribution changes following BCI intervention are regarded as direct electrophysiological evidence of motor system plasticity reorganization (Gangadharan et al., 2024; Remsik et al., 2019). Characteristic post-intervention ERD changes include: (i) increased ERD amplitude at ipsilesional C3/C4 electrodes, reflecting enhanced ipsilesional M1 engagement; (ii) spatiotemporally more focused distribution of ERD toward the contralateral motor cortex, indicating normalization of cortical reorganization; and (iii) significant correlation between the degree of mu rhythm desynchronization and behavioral motor improvement (Remsik et al., 2019).

Cortical synchrony analysis reveals higher-order network-level changes. Lee et al. (2022) employed weighted phase-lag index (wPLI) and directed transfer function (DTF) to analyze network properties during consecutive motor imagery in stroke patients, finding that a model predicting FMA upper-limb scores achieved an extraordinary *R*^2^ of 0.97 (RMSE = 1.68), indicating that network topology features are more sensitive motor function prediction biomarkers than single EEG indices. Mane et al. (2019) identified intervention-specific prognostic biomarkers—brain symmetry index (BSI) and power ratio index (PRI)—that distinguish BCI treatment from combined tDCS-BCI treatment, providing an objective basis for individualized treatment selection.

### 4.2 Neuroimaging evidence

Functional MRI (fMRI) provides the highest spatial resolution evidence of BCI-induced cortical reorganization. Yuan Z. et al. (2021) found in combined EEG-fMRI studies that BCI training significantly enhanced functional connectivity between the ipsilesional M1 and SMA in chronic stroke patients, and that this enhancement correlated significantly with FMA score improvement, with interhemispheric-level evidence of increased functional integration of the ipsilesional over the contralesional hemisphere. Caria et al. (2020) confirmed morphofunctional remodeling of the motor neuron system in severely affected chronic stroke patients following BMI intervention, including gray matter plasticity changes and structural reorganization of functional networks.

Rodríguez-García et al. (2025) published what is currently the most comprehensively evaluated neuroplasticity BCI study, simultaneously assessing changes in cortical activity, gray matter volume, and white matter fiber tracts. Results demonstrated that BCI treatment not only modified cortical activation patterns but also induced microstructural gray matter thickening around the ipsilesional motor area and increases in corticospinal tract white matter fractional anisotropy (FA), providing multi-level, multimodal structural–functional plasticity evidence.

### 4.3 TMS-based cortical excitability measures

TMS-evoked MEPs are the most direct neurophysiological index of corticospinal tract integrity and cortical excitability. MEP amplitude increases reflect elevated corticospinal tract excitability, whereas changes in the cortical silent period (CSP) are associated with the functional state of cortical inhibitory neurons (GABA-mediated) (Belardinelli et al., 2017).

Jochumsen et al. (2019) systematically compared EEG-triggered versus EMG-triggered electrical stimulation effects on corticospinal plasticity, finding that both triggers produced 62–73% MEP amplitude increases immediately post-intervention, maintained at 72–79% at 30 min post-intervention, confirming the stability of timing-paired corticospinal plasticity induction. Kim et al. (2022) demonstrated that combined multimodal BCI intervention—incorporating action observation and electrical stimulation—significantly enhanced corticospinal excitability, with MEP amplitude improvements superior to action observation or electrical stimulation alone, providing TMS evidence of multimodal synergistic effects.

### 4.4 Multimodal versus unimodal feedback: mechanistic comparison

Evidence for the mechanistic superiority of multimodal over unimodal feedback is accumulating. Shu et al. (2019) provided direct comparative evidence: under the “tactile stimulation plus motor intent” (TS-MA) condition, cortical motor-related activation (MRCA) in alpha–beta frequency bands was significantly higher than under motor intent alone (MA) or tactile stimulation alone (TS), and online BCI accuracy increased from 74.5% (MA) to 85.1% (TS-MA; *p* < 0.001), demonstrating that integration of sensory afference exerts a synergistic augmentation effect on cortical plasticity induction.

**Figure 3:**
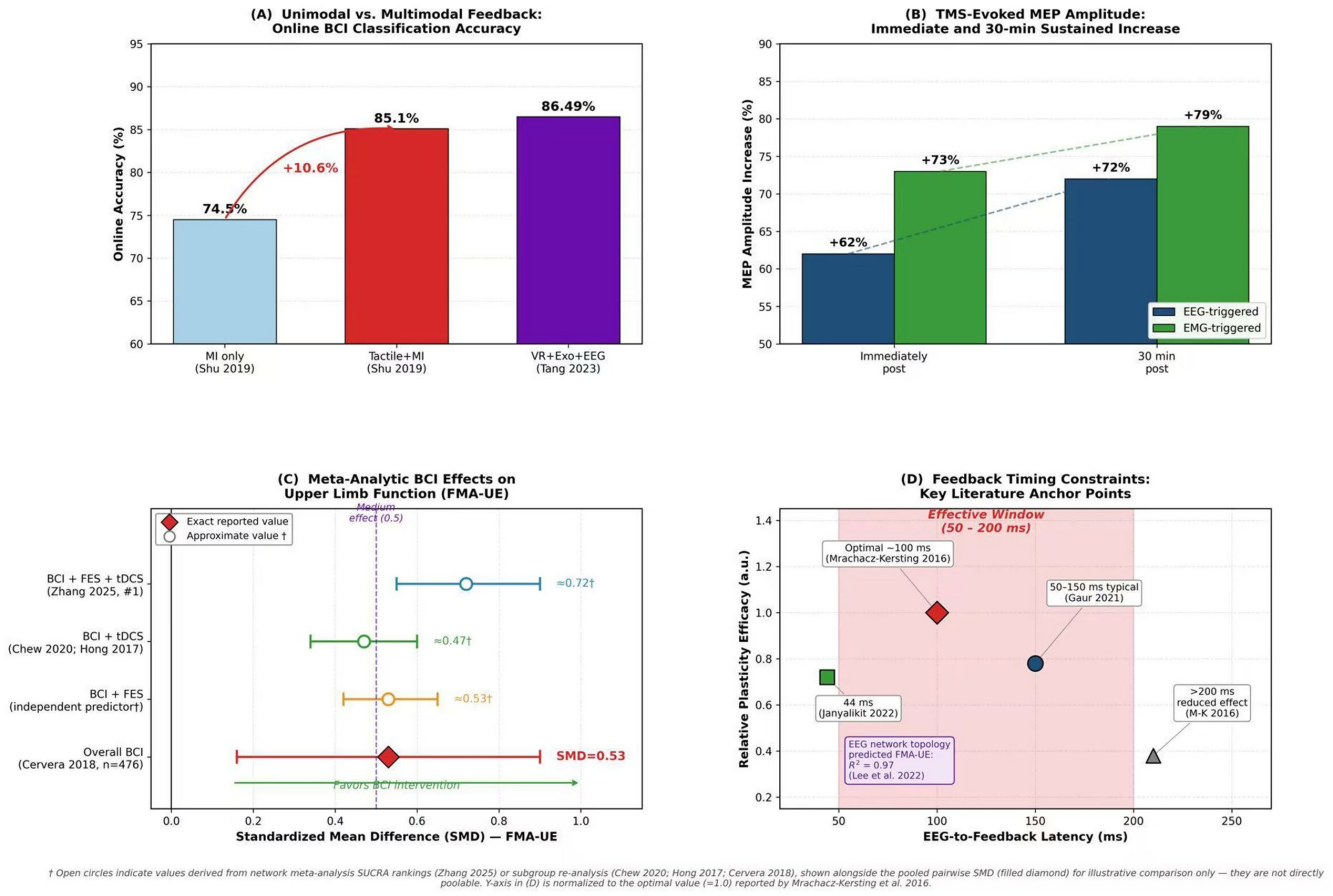
Key quantitative evidence for multimodal closed-loop BCI: Values directly reported in original publications.

**Figure 4:**
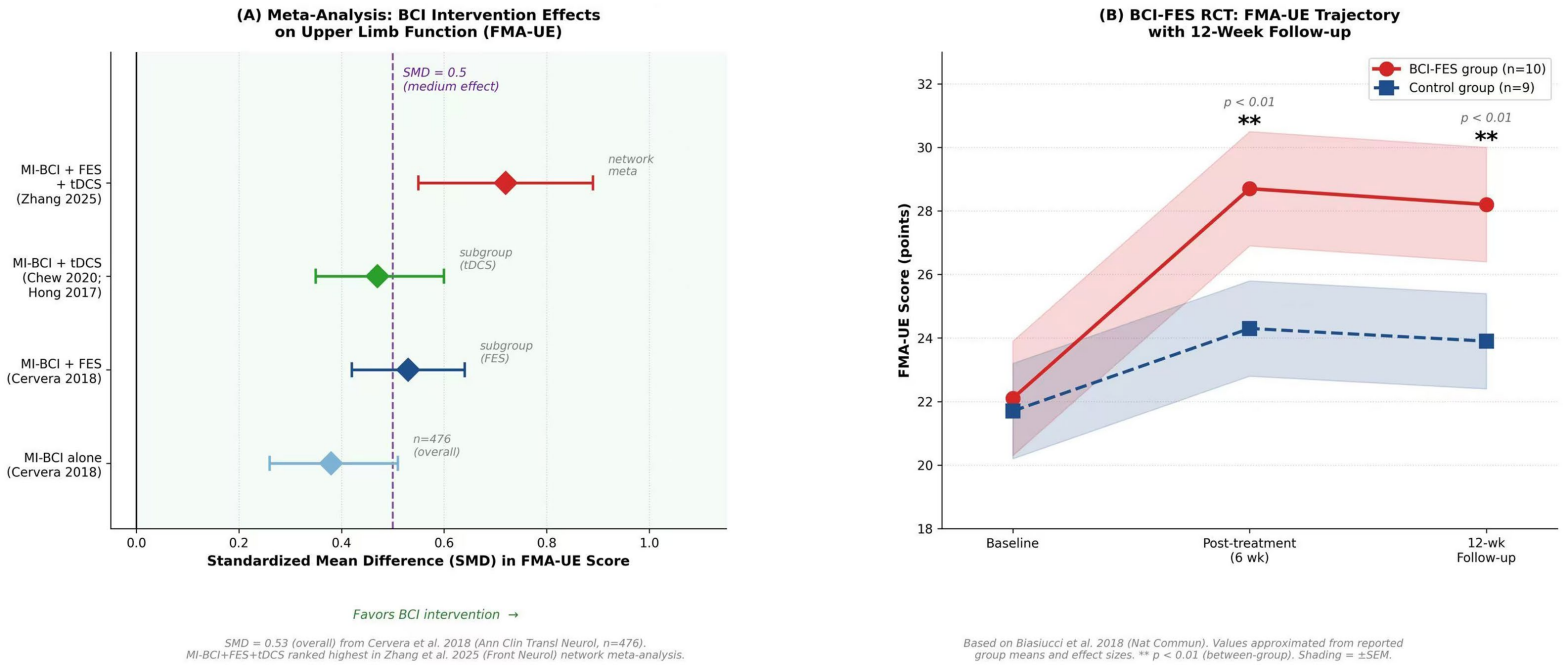
Clinical evidence: Meta-analytic effects and RCT outcomes of BCI-based stroke rehabilitation.

Rungsirisilp and Wongsawat (2022) and Rungsirisilp et al. (2023) compared the effects of combined action observation plus motor imagery (AOMI-BCI) versus motor imagery alone (MI-BCI) on upper-limb function recovery in chronic stroke patients. The AOMI group was significantly superior to the MI group on FMA-UE scores (*p* < 0.05), ipsilesional sensorimotor ERD amplitude, and online classification accuracy. From a meta-analytic perspective, Cervera et al. (2018)—synthesizing data from 476 patients—reported an upper-limb functional improvement effect size (standardized mean difference, SMD) of 0.53 (medium effect) for BCI interventions, with FES somatosensory feedback and high training dose identified as independent predictors of efficacy. Zhang et al. (2025)—in a network meta-analysis comparing MI-BCI, MI-BCI plus FES, MI-BCI plus tDCS, and other combined strategies—found that the MI-BCI plus FES plus tDCS triple combination ranked highest in FMA upper-limb score improvement. Key quantitative evidence supporting the superiority of multimodal over unimodal feedback is summarized in Figure 3. The meta-analytic standardized mean differences across BCI intervention strategies and the FMA-UE trajectory from the landmark BCI-FES RCT are presented in Figure 4.

The mechanistic superiority of multimodal over unimodal feedback can be understood at three levels of analysis. At the peripheral level, multimodal feedback simultaneously engages multiple afferent pathways—proprioceptive, tactile, visual, and kinesthetic—producing convergent sensory inputs to the sensorimotor cortex that are individually insufficient but collectively capable of activating the full complement of Hebbian synaptic remodeling mechanisms. At the cortical level, the concurrent activation of S1, M1, SMA, and parietal areas through multimodal afference strengthens inter-regional functional connectivity within the ipsilesional motor network more effectively than any single modality. At the network level, multimodal feedback engages both bottom-up sensory-driven plasticity and top-down motor intention-driven plasticity simultaneously, creating a bidirectional reinforcement of corticospinal synaptic efficacy that neither modality achieves independently.

It is important to note that the key determinant of neuroplasticity induction efficacy is not the number of feedback modalities per se, but rather the precision of temporal synchrony between motor intent signals and somatosensory afferent feedback. FES-based somatosensory feedback produces stronger and more consistent neuroplasticity effects than VR-only feedback primarily because peripheral nerve stimulation delivers afferent input with millisecond-level temporal precision, whereas visual feedback through VR involves additional cortical processing latency. Furthermore, the superiority of active BCI triggering over passive stimulation—consistently demonstrated across studies—underscores that the causal chain from motor intention to sensory consequence is itself a critical neuroplasticity driver, independent of the specific feedback modalities employed.

## 5 Key factors influencing neuroplasticity induction efficacy

### 5.1 Feedback contingency

Contingency—the temporal association between sensory feedback and motor intent (cortical discharge)—is the primary condition for activating Hebbian plasticity and the core mechanism distinguishing “active BCI closed-loop” from “passive stimulation” in terms of outcome. Biasiucci et al. (2018) precisely designed “intent-triggered” versus “randomly triggered” control conditions in their RCT, demonstrating that only intent-dependent FES triggering produced significant and sustained neuroplasticity effects, whereas random triggering produced no significant changes, directly verifying the necessity of contingency.

Dalgaard et al. (2026) further investigated the specificity of pairing afferent and efferent activity, finding that pairing specific sensory afferent modalities with specific motor commands induced more regionally specific cortical plasticity, while non-specific pairing was significantly less effective, indicating that the quality of temporal synchrony—not merely temporal proximity, but also modality matching—is equally important. Jochumsen et al. (2013) compared self-paced online BCI and cue-based offline BCI for neuroplasticity induction, finding that the self-paced paradigm induced more stable LTP-like cortical plasticity effects owing to better preservation of the authentic temporal structure of motor intent.

### 5.2 Feedback temporal synchrony

Under the prerequisite of guaranteed contingency, the absolute duration of feedback latency is a critical engineering constraint. Mrachacz-Kersting et al. (2016) demonstrated that approximately 100 ms post-MRCP peak represents the optimal stimulation timing window for Hebbian plasticity induction; latencies exceeding 200 ms significantly reduce plasticity effects by falling outside the STDP temporal window. This establishes a hard engineering design principle: the total end-to-end latency of the BCI pipeline—from neural signal acquisition to feedback delivery—must be maintained below 200 ms, with a target of approximately 100 ms for optimal neuroplasticity induction.

A systematic analysis of latency sources across the BCI pipeline reveals the following approximate contributions under typical implementation conditions: (1) signal acquisition buffer: 20–30 ms, determined by the EEG sampling rate and buffer size; (2) preprocessing and artifact rejection: 10–20 ms, including bandpass filtering, common average referencing, and epoch extraction; (3) feature extraction: 10–15 ms, including ERD/ERS power estimation or MRCP template matching; (4) classification and intent detection: 5–50 ms, highly dependent on model complexity—lightweight models such as EEGNet require as little as 5 ms, whereas deep Transformer architectures may require 30–50 ms on standard hardware; (5) communication and trigger transmission: 2–5 ms over USB or wireless protocols; and (6) FES activation and peripheral nerve conduction: 5–10 ms. Summing these components, well-optimized systems can achieve total end-to-end latencies of 50–130 ms, falling within the effective neuroplasticity induction window, while poorly optimized systems may exceed 200 ms and consequently fail to reliably activate Hebbian mechanisms.

Several system-level engineering strategies have been demonstrated to reduce pipeline latency without sacrificing decoding accuracy. First, hardware-accelerated inference using field-programmable gate arrays (FPGAs) or dedicated edge AI processors can reduce classification latency by an order of magnitude compared to general-purpose CPU-based implementations. Second, sliding-window prediction strategies—in which the classifier operates continuously on overlapping EEG epochs—substantially reduce effective detection latency (Saideepthi et al., 2023; Gaur et al., 2021). Third, asynchronous trigger architectures eliminate the additional latency introduced by synchronous polling loops. Fourth, lightweight model architectures such as EEGNet achieve competitive classification accuracy with minimal computational overhead, making them particularly suitable for portable home-based BCI systems.

The minimum reported end-to-end latency in the BCI literature to date is 44 ms, achieved by Janyalikit and Ratanamahatana (2022) using a time-series shapelet approach for self-paced ankle dorsiflexion detection. This benchmark establishes an aspirational target for next-generation closed-loop BCI hardware-software co-design. It should be noted that latency optimization must be balanced against classification accuracy, as aggressive latency reduction through model simplification may increase false positive rates and thereby compromise feedback contingency. The optimal operating point on the latency-accuracy trade-off curve is likely patient-specific, suggesting that adaptive latency management represents a valuable direction for personalized closed-loop BCI design.

### 5.3 Stroke phase and neuroplasticity potential

Stroke phase exerts an important influence on BCI treatment outcomes: the acute phase (<1 month) offers the highest endogenous plasticity potential, but patient instability and weak available EEG signals are limiting factors; the subacute phase (1–6 months) represents the optimal intervention window, coinciding with the spontaneous recovery period; chronic-phase patients (>6 months) have reduced neuroplasticity but can still achieve functional improvement through long-term systematic training (He et al., 2025; Yuan Z. et al., 2021).

Wu et al. (2020) specifically assessed cerebral functional network changes following BCI training in the subacute phase, finding that changes in connectivity between the default mode network and motor network were most pronounced in subacute patients, indicating that this phase represents the most active period of neural “network reconstruction.” Liu et al. (2026) developed a gait phase encoding sequence electrical stimulation-enhanced motor imagery (SES-MI) paradigm specifically targeting the problem of weak lower-limb MI activation in stroke patients, achieving 81.30% accuracy in 2-class classification, with SES-MI inducing ERD amplitudes 115% higher than those evoked by text-cued MI alone, demonstrating the compensatory value of external sensory augmentation for patients with weak MI signals.

### 5.4 Training dose, individual differences, and prognosis prediction

Training dose (total training duration, session length, training frequency) is an important parameter influencing BCI rehabilitation outcomes, but standardized guidelines are currently lacking. Foong et al. (2020) demonstrated in a multi-center trial that a BCI training regimen of three sessions per week, 90 min per session, for 6 weeks produced significant FMA score improvements that were sustained at 24-week follow-up, suggesting that this regimen achieves the minimum effective dose for inducing long-term plasticity. Yuan Z. et al. (2021) reported that 24 sessions of BCI-cycling training produced a mean FMA lower-limb score improvement of 4.5 points in the experimental group (versus 2.1 points in controls; *p* = 0.022).

Individual differences—including lesion location, baseline neuroplasticity level, and MI ability—are the core challenge in BCI efficacy prediction. Lin et al. (2021) proposed a CNN prognostic model based on EEG from the first BCI training session that utilized functional connectivity and power spectral density features to predict 2-week rehabilitation outcomes in 11 stroke patients, achieving *R*^2^ = 0.98, suggesting that baseline EEG data can be used for early patient stratification. Shetty et al. (2026) and Fan et al. (2023) further confirmed that brain entropy and signal complexity can serve as objective neuroplasticity biomarkers, potentially applicable to real-time rehabilitation progress monitoring.

### 5.5 Patient heterogeneity and inter-individual variability

Patient heterogeneity represents one of the most significant and underappreciated challenges in translating multimodal closed-loop BCI rehabilitation from controlled research settings to routine clinical practice. Stroke survivors constitute an exceptionally diverse population across multiple dimensions that directly influence BCI performance and neuroplasticity induction efficacy.

Lesion characteristics exert a foundational influence on BCI rehabilitation potential. Lesion location determines which cortical and subcortical motor networks are disrupted: damage to the primary motor cortex (M1) or corticospinal tract (CST) directly impairs the neural substrate targeted by BCI intervention, whereas subcortical lesions sparing M1 may preserve greater residual cortical plasticity potential. Lesion volume correlates inversely with baseline corticospinal tract integrity, as assessed by diffusion tensor imaging fractional anisotropy (FA) of the posterior limb of the internal capsule—a biomarker that has been shown to predict upper-limb motor recovery potential (Rodríguez-García et al., 2025).

Baseline neuroplasticity capacity varies substantially across individuals and is influenced by age, pre-stroke cognitive reserve, and the integrity of non-lesioned motor network components. Older patients typically exhibit reduced synaptic plasticity potential owing to age-related decreases in NMDA receptor density and BDNF expression, potentially limiting the magnitude of LTP inducible by BCI intervention (Chen et al., 2025). However, Chen et al. (2025) demonstrated that sustained long-term BCI–FES intervention can induce continuous neuroplasticity even in elderly stroke patients, suggesting that reduced baseline plasticity capacity can be partially compensated by increased training duration and intensity.

Motor imagery (MI) ability varies enormously across individuals, with a substantial proportion classified as “BCI-inefficient” (low BCI performance, LBP). In stroke patients, MI ability is further compromised by cortical lesions, attentional deficits, and reduced proprioceptive feedback. Zhang et al. (2023) addressed this challenge through an adaptive BCI system that dynamically adjusts stimulation parameters based on real-time BCI performance, enabling LBP patients to participate in and benefit from FES rehabilitation. Liu et al. (2026) demonstrated that gait phase encoding electrical stimulation can augment lower-limb MI signals by 115% compared to text-cued MI alone, illustrating the potential of external sensory augmentation to compensate for weak endogenous MI signals.

Addressing patient heterogeneity requires a shift toward precision medicine approaches. Early biomarker-based patient stratification—using baseline EEG features (Lin et al., 2021), CST integrity (FA values), and MI ability assessments—can identify patients most likely to benefit from specific BCI configurations. The CNN-based prognostic model proposed by Lin et al. (2021), achieving *R*^2^ = 0.98 for 2-week rehabilitation outcome prediction, exemplifies the potential of data-driven stratification. Brain entropy and signal complexity metrics proposed by Shetty et al. (2026) offer complementary real-time monitoring capabilities for dynamic adjustment of BCI parameters throughout the rehabilitation course.

## 6 Discussion

### 6.1 Multimodal signal fusion and decoding accuracy

Despite significant advances in deep learning for MI decoding, the low signal-to-noise ratio and high inter-individual variability of stroke patient EEG signals remain core bottlenecks constraining system practicality. Most existing algorithms perform well on healthy subject datasets, but their generalizability to real stroke patient data requires substantial improvement (Bhalerao and Pachori, 2025). Multimodal signal fusion (EEG plus EMG plus fNIRS), while theoretically offering complementary advantages, remains an open engineering challenge for the efficient joint decoding of multimodal features on embedded real-time platforms. For the data scarcity of EEG, generative adversarial networks (GANs) (Xu et al., 2021) and contrastive self-supervised learning (Wang and Li, 2025) have demonstrated potential for improving small-sample learning performance, but validation on real clinical data remains insufficient.

The generalizability challenge is further compounded by the fundamental difference between algorithm development and clinical validation workflows. Most deep learning decoders are developed and benchmarked on standardized datasets comprising healthy subjects or small chronic stroke cohorts under controlled laboratory conditions, then deployed in clinical settings with substantially different signal characteristics, noise environments, and patient populations. Bridging this gap will require prospective clinical validation studies specifically designed to evaluate decoder performance under ecologically valid conditions, including variable electrode impedance, movement artifacts, and fatigue-related signal drift across extended rehabilitation sessions.

Beyond motor rehabilitation, multimodal neural decoding architectures have demonstrated remarkable progress in adjacent BCI domains. Al-Radhi et al. (2025) proposed MiSTR, a multi-modal iEEG-to-speech synthesis framework integrating wavelet-based neural feature extraction with Transformer-based prosody prediction and a neural phase vocoder, achieving state-of-the-art speech intelligibility (Pearson correlation = 0.91). Although MiSTR employs intracranial EEG rather than scalp EEG, its cross-modal neural representation learning strategies—particularly the integration of temporal, spectral, and neurophysiological features—offer potentially transferable methodological insights for improving the robustness and generalizability of motor intent decoding in stroke rehabilitation contexts. Cross-domain integration between speech and motor BCI research represents a promising future direction.

### 6.2 Standardization of neuroplasticity assessment

Current literature employs highly diverse indices for evaluating BCI-induced neuroplasticity—including ERD/ERS amplitude, MEP amplitude, fMRI functional connectivity, and FA values—with considerable heterogeneity in measurement time points and analytical methods across studies, rendering cross-study comparisons difficult. Establishing a multidimensional standardized assessment framework incorporating electrophysiology (EEG/TMS), neuroimaging (fMRI/fNIRS), and clinical function (FMA/ARAT/Wolf Motor Function Test) represents an urgent need for the field’s normative development. Shetty et al. (2026), proposing brain entropy and complexity metrics, and Han et al. (2025), identifying neural biomarkers via explainable graph neural networks, represent development trajectories toward quantification and objectification of neuroplasticity indices.

A critical examination of the clinical efficacy evidence reveals several important methodological limitations. The majority of RCTs in this field involve small sample sizes (median *n* < 20), limiting statistical power and increasing the risk of inflated effect size estimates. Few studies employ active sham-controlled designs—in which control participants receive matched sensory stimulation without BCI contingency—making it difficult to isolate the specific contribution of closed-loop neural feedback. The landmark study by Biasiucci et al. (2018), which directly compared intent-triggered versus randomly triggered FES, remains the most rigorous demonstration of BCI-specific neuroplasticity effects and should serve as a methodological template for future RCT design.

### 6.3 Personalized adaptive closed-loop systems

The ideal closed-loop BCI system should dynamically adjust training parameters—task difficulty, stimulation intensity, and feedback modality weighting—based on the patient’s real-time neural state (fatigue, attentional level, learning progress), achieving truly individualized adaptive treatment. The majority of current systems still employ preset fixed parameters and lack closed-loop adaptive capacity based on neural feedback (Chowdhury et al., 2018; Wang et al., 2025b). The attention detection-based adaptive system developed by Lim et al. (2026) represents an important step toward this goal. Future development will need to integrate reinforcement learning (RL) technology to allow BCI systems to autonomously optimize training strategies based on individual neural responses, realizing the vision of “BCI as a neuroplasticity amplifier.”

### 6.4 Home-based deployment and long-term maintenance of effects

Translating multimodal closed-loop BCIs from the laboratory to the home environment is pivotal for achieving large-scale clinical deployment. Home-based systems require high user-friendliness, low calibration costs, robust noise tolerance, and wireless portable hardware design. Chowdhury et al. (2026) released the Neurestore benchmark dataset specifically targeting real-world noisy home environments, providing an important reference for standardized algorithm evaluation in this context. Rao et al. (2025) substantially lowered the usage threshold through the single-calibration strategy; the combination of low-density wearable EEG with lightweight decoding algorithms represents a feasible technical pathway for home-based multimodal BCIs. Evidence for long-term maintenance of effects (3–12 months or more follow-up) remains limited; Chen et al. (2025)—with their 4.5-year study—is an exception, and future research requires additional well-designed long-term follow-up RCTs.

### 6.5 Limitations of current evidence and future directions

#### 6.5.1 Sample size and statistical power

The most pervasive limitation across the BCI rehabilitation literature is small sample size. The majority of published RCTs involve fewer than 20 participants per group, providing limited statistical power to detect moderate effect sizes. Meta-analytic pooling by Cervera et al. (2018) across 476 patients provided the most statistically robust estimates to date (overall SMD = 0.53), but the high heterogeneity across included studies limits the precision of subgroup analyses. Future research requires adequately powered multicenter RCTs with pre-registered sample size calculations.

#### 6.5.2 Outcome measure heterogeneity

Substantial heterogeneity exists across studies in the selection, timing, and analytical methods applied to both neuroplasticity and clinical outcome measures. Establishing a standardized multidimensional assessment battery—incorporating at minimum one electrophysiological index (EEG ERD or TMS MEP), one neuroimaging index (fMRI functional connectivity or DTI FA), and one validated clinical outcome measure (FMA-UE)—assessed at standardized time points (baseline, post-treatment, 3-month follow-up, 12-month follow-up) should be adopted as a field-wide standard.

#### 6.5.3 Control condition design

The majority of BCI rehabilitation studies employ passive control conditions that do not adequately isolate the specific contribution of closed-loop neural feedback from non-specific therapeutic effects. Active sham-controlled designs, in which control participants receive matched sensory stimulation delivered non-contingently or at random intervals, are essential for establishing that neuroplasticity effects are specifically attributable to the Hebbian temporal contingency mechanism.

#### 6.5.4 Population representativeness

The predominance of chronic stroke populations (>6 months post-stroke) in published BCI rehabilitation studies limits the generalizability of findings to the subacute phase. Prospective studies specifically targeting the subacute phase, with longitudinal neuroimaging assessment, are urgently needed. Additionally, the underrepresentation of patients with severe motor impairment, aphasia, and significant cognitive deficits limits the applicability of current evidence.

#### 6.5.5 Offline-to-online generalization gap

The substantial performance gap between offline benchmark results and online clinical performance remains a critical unresolved challenge. Prospective validation of decoding algorithms under ecologically valid conditions—including variable electrode impedance, movement artifacts, fatigue-related signal drift, and real-world noise environments—is an essential prerequisite for safe and effective clinical translation.

#### 6.5.6 Long-term maintenance of effects

Evidence for the long-term maintenance of BCI-induced neuroplasticity beyond 6 months post-intervention remains limited. Whether the structural and functional plasticity changes induced by multimodal closed-loop BCI intervention are permanent, progressive, or subject to decay in the absence of continued training is a fundamental question with direct implications for rehabilitation program design. Large-scale, well-designed long-term follow-up RCTs extending to 12 months or beyond are an urgent priority.

#### 6.5.7 Future directions

Building on the limitations identified above, the highest-priority future research directions include: (1) reinforcement learning-based personalized adaptive closed-loop systems that autonomously optimize training parameters based on individual neural responses in real time; (2) multimodal biomarker-guided patient stratification frameworks integrating baseline EEG complexity, CST integrity, MI ability, and lesion characteristics; (3) multicenter large-sample RCTs with standardized outcome batteries, active sham controls, and long-term follow-up; and (4) fully home-deployable multimodal BCI rehabilitation platforms combining low-density wearable EEG, portable FES, and consumer-grade VR with single-calibration decoding algorithms and remote monitoring capabilities. A critical engineering design principle derived from this review: feedback latency must be maintained below 100 ms (ideally ~100 ms post-MRCP peak) to reliably activate STDP-based Hebbian plasticity; system designs exceeding 200 ms total latency should be considered outside the effective neuroplasticity induction window.

## 7 Conclusion

Multimodal closed-loop brain–computer interfaces, by precisely synchronizing motor intent signals with multimodal sensory feedback—FES somatosensory, VR visual, and exoskeleton proprioceptive—provide the intervention paradigm with the strongest theoretical grounding and clinical evidence for active induction of post-stroke neuroplasticity. Core mechanistic pathways include activity-dependent Hebbian synaptic remodeling via temporally paired LTP, restoration of ipsilesional sensorimotor cortical excitability with correction of interhemispheric inhibitory imbalance, and sensorimotor integration-driven cortical reorganization.

Converging evidence from electrophysiology (ERD/ERS, functional connectivity), neuroimaging (fMRI activation, gray and white matter plasticity), and TMS (MEP amplitude, corticospinal tract integrity) demonstrates that multimodal closed-loop BCIs can induce objectively measurable neuroplasticity changes in chronic, subacute, and a proportion of acute stroke patients; that multimodal synergistic effects are superior to unimodal interventions; and that BCI active triggering is superior to passive stimulation. Nevertheless, the field continues to confront three core challenges: generalizability of decoding algorithms to clinical patients, standardization of neuroplasticity assessment, and home-based deployment and long-term maintenance of effects. Future development should focus on constructing reinforcement learning-based personalized adaptive closed-loop systems and establishing evidence-based medicine through multicenter large-sample RCTs, thereby advancing multimodal closed-loop BCIs from the laboratory into routine clinical rehabilitation practice.
